# Case report: Cerebral syphilitic gumma: a case retrospective report of eight cases

**DOI:** 10.3389/fmed.2024.1448698

**Published:** 2024-11-27

**Authors:** Jun Liu, Wenjun Zhang, Qiuhua Jiang, Qianliang Huang, Xinyun Ye

**Affiliations:** ^1^Department of Neurosurgery, The 2nd Affiliated Hospital, Jiangxi Medical College, Nanchang University, Nanchang, Jiangxi, China; ^2^Department of Neurosurgery, The Ganzhou Affiliated Hospital, Jiangxi Medical College, Nanchang University, Ganzhou, Jiangxi, China; ^3^Rehabilitation Medicine Department, The Ganzhou Affiliated Hospital, Jiangxi Medical College, Nanchang University, Ganzhou, Jiangxi, China

**Keywords:** cerebral syphilitic gumma, *Treponema pallidum*, case series, clinical feature, diagnostic and treatment strategies

## Abstract

**Background:**

Cerebral syphilitic gumma (CSG), a rare manifestation of neurosyphilis, presents characteristics akin to intracranial tumors, often leading to clinical misdiagnosis.

**Objective:**

This study aimed to summarize the clinical experience in diagnosing and treating CSG.

**Materials and methods:**

The present study conducted a retrospective analysis of clinical data, encompassing the baseline characteristics, clinical presentation, diagnosis, treatment, and prognosis of eight patients with CSG who were treated and diagnosed by our institution.

**Results:**

The median age at the initial diagnosis was 57.5 years, comprising six males and two females. The predominant clinical manifestations included headaches in eight cases, motor and sensory disorders in four cases, epileptic seizures in one case, and dysarthria in one patient. The serum *Treponema pallidum* haemagglutination assay (TPHA) and toluidine red unheated serum test (TRUST) yielded positive in all eight patients. The contrast-enhanced magnetic resonance imaging (MRI) revealed irregular ring-shaped enhancement of lesions in three patients, while nodular enhancement of lesions was observed in five patients. In terms of treatment, seven patients underwent surgery. The postoperative pathological tissue showed granulomatous inflammation. Six patients exhibited an elevated protein concentration in their cerebrospinal fluid (CSF), while two patients demonstrated a reduced CSF glucose level. Additionally, positive results were obtained for both CSF TPHA and TRUST in all eight patients. The clinical diagnosis of CSG was confirmed in eight patients who exhibited notable clinical improvement following penicillin treatment. Subsequent reevaluation of the imaging findings demonstrated complete resolution of the enhanced lesions.

**Conclusion:**

In clinical practice, CSG should be considered for patients with intracranial lesions and positive serum syphilis antibodies. Timely and accurate diagnosis enables patients with CSG to achieve a more favorable prognosis through active anti-syphilis treatment.

## Introduction

Neurosyphilis is an infectious disease caused by the invasion of the nervous system by the *Treponema pallidum*. The nervous system, apart from the mucocutaneous membranes, exhibits the highest susceptibility to *Treponema pallidum* infection (1). Cerebral Syphilitic gumma (CSG) represents a distinct subtype of neurosyphilis, yet the precise incidence rate remains inadequately documented in previous literature. Only Drago et al. have explicitly reported that CSG accounts for ~3.5% of all neurosyphilis cases (2). Due to the low incidence of CSG and the non-specific nature of its clinical symptoms, many physicians lack sufficient expertise in diagnosing this condition, leading to frequent oversight of it as a differential diagnosis. It is easy to be misdiagnosed as a brain tumor and undergo surgical resection or craniotomy biopsy (2–5). Therefore, enhancing the understanding of CSG is of practical significance in terms of improving the accuracy of its diagnosis.

This study retrospectively analyzed the clinical data of eight patients with CSG who were diagnosed and treated between January 2014 and August 2023 and integrated it with relevant literature to provide a comprehensive overview of diagnostic approaches and treatment strategies, aiming to offer valuable clinical insights.

## Materials and methods

### Data collection

We conducted a comprehensive search in the hospital's electronic medical record database using the specific keyword “syphilitic cerebral gumma” and identified a total of eight cases involving intracranial lesions from January 2014 to August 2023. Subsequently, we systematically gathered comprehensive clinical data, including patients' medical history, laboratory test results for serum and cerebrospinal fluid (CSF) samples, head imaging examinations, pathological tissue findings, and treatment modalities employed, as well as the corresponding outcomes.

### Patient characteristics and clinical findings

A total of eight CSG cases were included in the study (Table 1), comprising six males and two females. The age range was 38–69 years, with a median age of 57.5 years. All patients presented with varying degrees of headache symptoms, while four patients exhibited symptoms of unilateral limb numbness or weakness, one patient experienced epileptic seizures, and one patient manifested unclear speech symptoms. None of the patients had an acute onset or rapid progression. Upon admission, three male patients reported a history of syphilis. However, it is unknown whether their treatment was standardized and sufficient. None of the female patients were aware of their syphilis status. All patients exhibited negative serum HIV antibodies and positive serum *Treponema pallidum* hemagglutination assay (TPHA). The results of the toluidine red unheated serum test (TRUST) ranged from 1:1 to 1:64, with three cases exhibiting TRUST values < 1:8 and five cases demonstrating values ranging from 1:16 to 1:64.

**Table 1:** Summary of patient characteristics and clinical findings.

**Patients**	**Gender**	**Age**	**Clinical symptoms**	**Medical history**	**TRUST of serum**	**TPHA of serum**	**Treatment modality**	**Histopathological examination**	**Follow-up result**	**Prognosis**
1	Male	38 years	Headache, numbness of limbs	History of Syphilis and naot standardized treatment	1:4	+	Surgical treatment and treatment with penicillin	Syphilitic gumma	The lesions disappeared after 18 months	Complete remission
2	Male	59 years	Headache	History of Syphilis	1:32	+	Surgical treatment and treatment with penicillin	Syphilitic gumma	The lesions disappeared after 12 months	Complete remission
3	Male	48 years	Headache, numbness and weakness of limbs	Denied a history of Syphilis and not standardized treatment	1:2	+	Surgical treatment and treatment with penicillin	Syphilitic gumma	The lesions disappeared after 1 month	Complete remission
4	Female	64 years	Headache, numbness and weakness of limbs	Uncertain	1:64	+	Surgical treatment and treatment with penicillin	Syphilitic gumma	The lesions disappeared after 9 months	Partial remission
5	Male	44 years	Headache, epilepsy	History of Syphilis and not standardized treatment	1:32	+	Surgical treatment and treatment with penicillin	Syphilitic gumma	The lesions disappeared after 3 months	Partial remission
6	Male	69 years	Headache	Denied a history of Syphilis	1:16	+	Surgical treatment and treatment with penicillin	Syphilitic gumma	The lesions disappeared after 6 months	Complete remission
7	Male	65 years	Headache, glossolalia	Denied a history of Syphilis	1:8	+	Surgical treatment and treatment with penicillin	Syphilitic gumma	The lesions disappeared after 3 months	Complete remission
8	Female	56 years	Headache, weakness of limbs	Uncertain	1:64	+	Treatment with penicillin	/	The lesions disappeared after 2 months	Complete remission

### Radiologic findings

**Figure 1:**
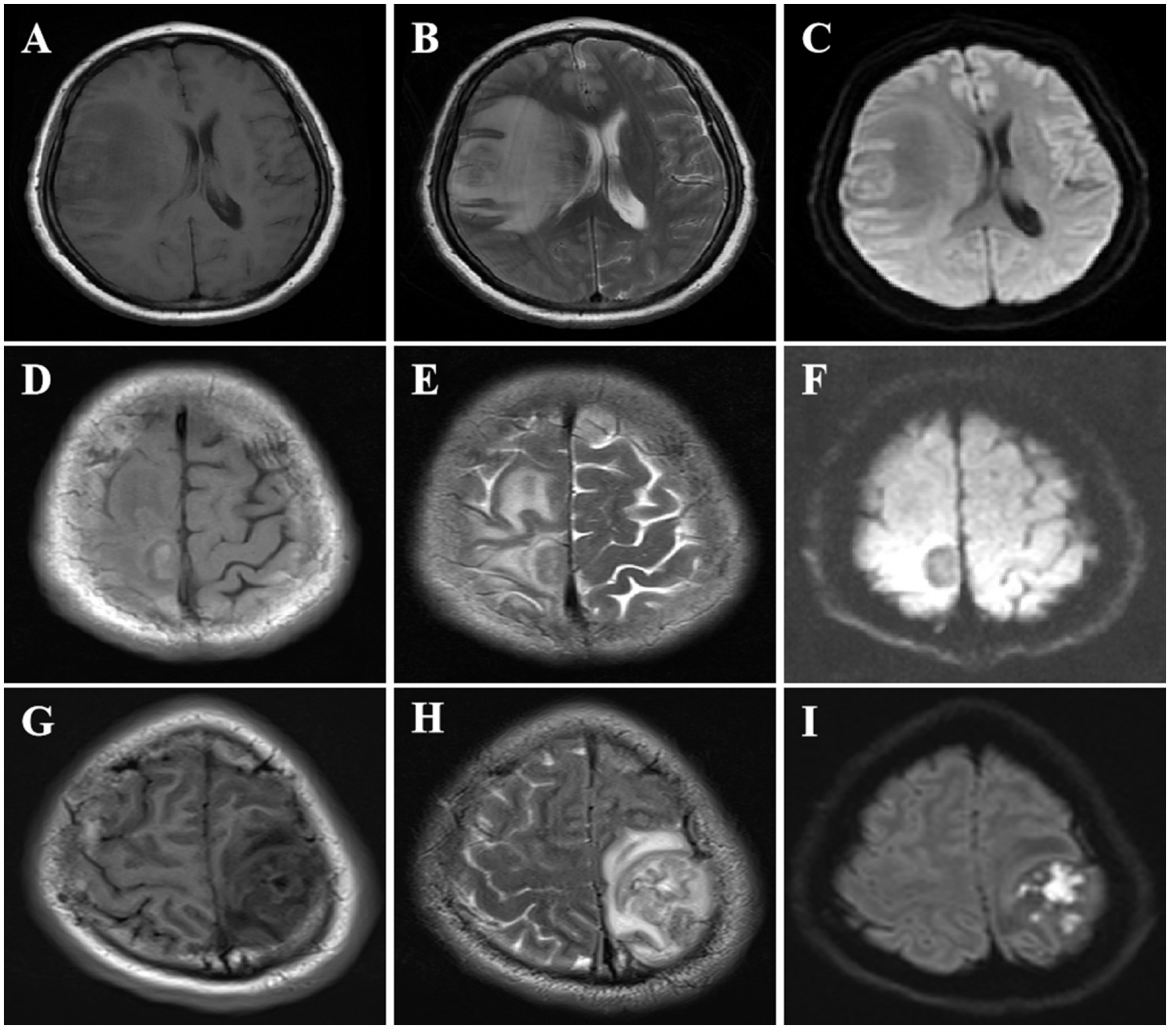
MRI imaging features of CSG. MRI imaging features of SCG. **(A–C)** The right frontotemporal lobe exhibited a nodular mass characterized by low signal on T1WI, high signal on T2WI, and restricted diffusion with hypointensity on DWI. **(D–F)** A well-defined round mass was observed in the right frontal lobe adjacent to the falx cerebri, exhibiting slightly low signal on T1WI, high signal on T2WI, and high signal surrounding the lesion on DWI. **(G–I)** The left parietal lobe exhibited a mass occupying lesion with slightly decreased signal intensity on T1WI, iso-high signal intensity on T2WI, and high signal intensity on DWI, iso-high signal intensity surrounding the lesion.

**Figure 2:**
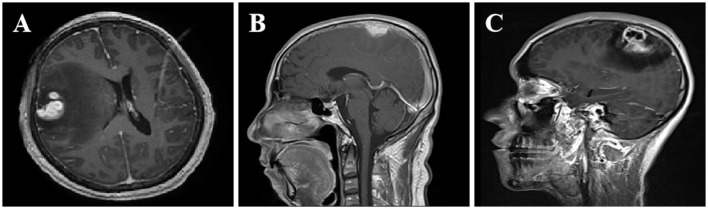
Enhanced MRI imaging features of CSG. MRI imaging features of SCG. **(A)** After contrast enhancement, the lesions in the right frontotemporal lobe showed nodular enhancement. **(B)** The right frontal lobe lesions demonstrated homogeneous and prominent enhancement upon contrast-enhanced scan, accompanied by thickening and enhancement of the adjacent meninges. **(C)** The lesions in the left parietal lobe showed uneven ring-like enhancement after contrast enhancement, accompanied by thickening and enhancement of the adjacent meninges.

The information derived from the head magnetic resonance imaging (MRI) scan of all patients is shown in Table 2. The imaging examinations of all eight patients revealed a solitary lesion, with the distribution as follows: four cases in the frontal lobe, two cases in the parietal lobe, one case in the temporal lobe, and one case in the frontotemporal lobe. The head MRI examinations were performed on all patients, revealing lesions with low or slightly low signal on T1-weighted imaging (T1WI), high or slightly high signal on T2-weighted imaging (T2WI), and mixed high and low signal on diffusion-weighted imaging (DWI) (Figures 1A–I). All lesions were surrounded by a high-signal edematous area. The lesions exhibited irregular thick-walled annular enhancement or nodular enhancement on T1 contrast-enhanced MRI, accompanied by thickening and enhancement of the adjacent meninges (Figures 2A–C).

**Table 2:** MRI imaging features for all patients.

**Patients**	**Pathogenic site**	**Magnetic resonance imaging of head**			
		**T1WI**	**T2WI**	**DWI**	**Enhanced image**
1	Left parietal lobe	Low signal intensity	Slightly higher signal	High signal intensity	Irregular ring of enhancement involving the adjacent dura mater, with non-enhancement areas
2	Right frontal lobe	Slightly lower signal	High signal intensity	High signal intensity	Homogeneous and marked enhancement with adjacent meninges
3	Left frontal lobe	Slightly lower signal	High signal intensity	High signal intensity	Irregular ring of enhancement involving the adjacent dura mater, with non-enhancement areas
4	Left parietal lobe	Low signal intensity	Slightly higher signal	High and low mixed signals	Significant enhancement and thickening enhancement of the adjacent meninges, with non-enhancement areas in the interior
5	Right temporal lobe	Low signal intensity	Slightly higher signal	High signal intensity	Marked enhancement, partial meningeal enhancement
6	Right frontal lobe	Slightly lower signal	Slightly higher signal	Isointensity	Marked and heterogeneous enhancement involving the adjacent meninges, with non-enhancement areas in the interior
7	Left frontal lobe	Slightly lower signal	Slightly higher signal	High signal intensity	Irregular annular enhancement involving the adjacent dura mater
8	Right frontotemporal lobe	Low signal intensity	High signal intensity	Low signal intensity	Homogeneous and marked enhancement with thickening and enhancement of the adjacent meninges

### Treatment and outcomes

**Figure 3:**
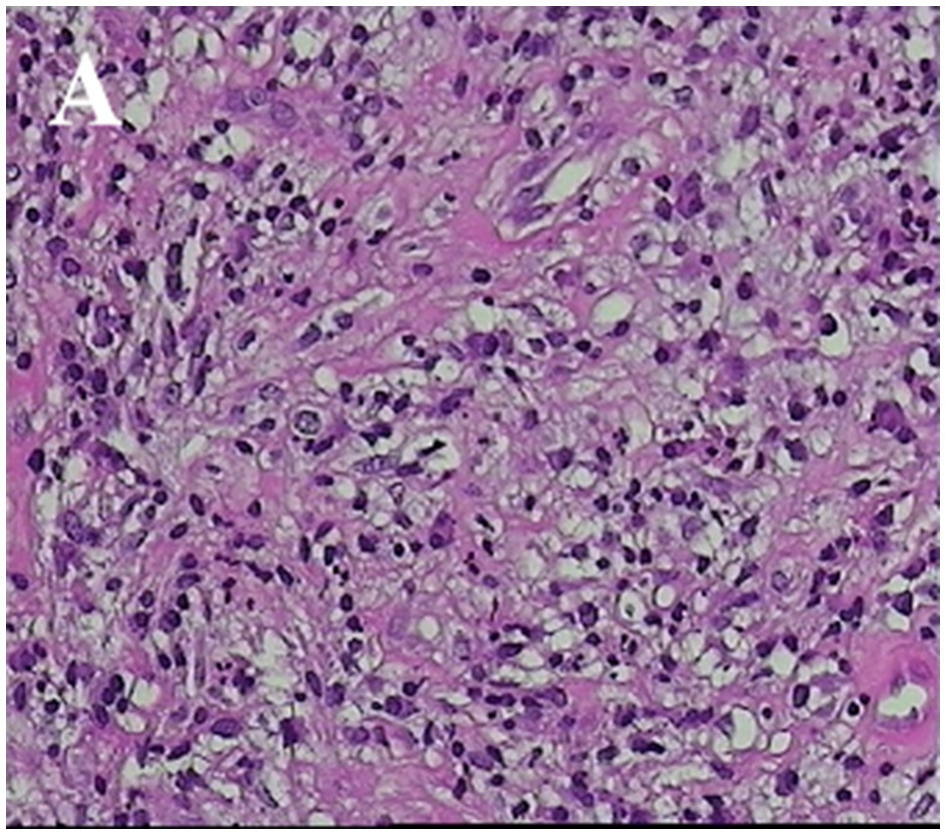
Pathological characteristics of CSG. **(A)** The specimen exhibited partial brain tissue, characterized by neuronal degeneration and necrosis, as well as localized gliosis. Infiltration of numerous lymphocytes, plasma cells, and a limited number of neutrophils was observed (HE; × 200 magnification).

Seven patients who were initially diagnosed with brain tumors underwent microsurgical craniotomy to remove the lesions, and some patients received navigational guidance before surgery and B-ultrasound assistance during the procedure for precise localization of the lesion. During the surgical procedure, a gray-yellow lesion was observed, exhibiting a relatively firm texture and unclear boundaries with the surrounding area. Additionally, there was evidence of edema in the surrounding brain tissue. The lesions were poor blood supply, and partial adhesion with the dura mater in some cases. During the operation, a routine fast frozen pathological examination of the lesion tissue was performed, which indicated an inflammatory lesion. Consequently, the surgical resection plan was adjusted. The microscopic examination of the lesion tissue revealed the presence of rubbery nodules within the lesion, characterized by necrotic foci and multiple giant cell reactions, these nodules were surrounded by infiltration of inflammatory cells and proliferation of fibrotic tissue (Figure 3A). The postoperative pathological examination revealed the lesion as a granulomatous inflammation. CSF examinations were conducted post-surgery in seven patients, while in another case, CSF was examined immediately following the clinical diagnosis of CSG (Table 3). The CSF pressure ranged from 240 to 320 mmHg (1 mmHg = 0.133 kPa) in eight patients, indicating varying degrees of elevated intracranial pressure. The concentration of CSF protein ranged from 0.374 to 1.286 g/L, with elevated levels (>0.45 g/L) observed in six patients and normal concentrations found in two patients. The glucose levels in the CSF ranged from 2.8 to 4.3 mmol/L, with two cases exhibiting low values and the remaining patients showing normal levels. The white blood cell (WBC) count in the CSF exhibited varying degrees of elevation, with positive results observed for both TPHA and TRUST across all patients. The diagnosis of CSG was ultimately confirmed in all patients based on CSF findings and pathological examinations. The treatment plan comprises a total of 18 million units of intravenously administered benzylpenicillin sodium, given twice daily over a continuous period of 2 weeks. The patients in this cohort exhibited a favorable prognosis after treatment, as evidenced by varying degrees of improvement in clinical symptoms and signs upon discharge. All patients were followed up for 3 months to 1.5 years after treatment, and the follow-up head computerized tomography (CT) scans showed varying degrees of softening focus formation.

**Table 3:** The pretention of CSF results.

**First CSF examination**					**Final CSF examination**				
**TRUST**	**TPHA**	**Protein content, mg/L**	**White blood cell count, 10** ^6^	**Glucose levels, mmol/L**	**TRUST**	**TPHA**	**Protein content, mg/L**	**White blood cell count, 10** ^6^	**Glucose levels, mol/L**
1:4	+	1,063	11.2	5.21	1:2	+	388	4.6	2.3
1:16	+	720	9.6	2.43	1:4	+	289	3.8	3.23
1:2	+	435	10.3	3.56	1:1	-	251	2.3	3.3
1:32	+	1,286	7.1	4.8	1:4	+	353	3.2	3.8
1:4	+	374	10	2.8	1:2	+	287	4.2	2.83
1:8	+	656	6.9	3.77	1:2	-	198	3.9	2.68
1:4	+	776	9.1	4.49	1:1	-	219	4	3.1
1:16	+	865	6.8	4.36	1:4	+	236	3.3	3.35

## Discussion

CSG are terminal-stage syphilitic lesion in the brain parenchyma or meninges, it is an inflammatory granulomatous lesion caused by the spread of syphilis spirochetes into the brain (1, 2). These lesions can occur anywhere in the intracranial region, and clinically they are most commonly found on the convex side of the brain (2, 6, 7). The distribution of lesions in this cohort predominantly involved the frontal and parietal lobar cortex, exhibiting a solitary pattern consistent with the reported literature findings. The clinical symptoms of CSG resemble those observed in other intracranial space-occupying lesions, with the presence of edema surrounding the lesion being a common feature, thereby leading to cranial hypertension (2, 8). Generally, patients with CSG do not exhibit fever. We hypothesize that this may be attributed to the development of a thick-walled capsule around the gumma following secondary immune reactions, effectively isolating it from the subarachnoid space and CSF. Consequently, clinical examinations typically do not reveal meningeal inflammatory reactions such as signs of meningeal irritation. In the examination of all eight patients in our cohort, no significant abnormalities were found in their body temperature, and no meningeal irritation symptoms were observed. CSG is a secondary manifestation of syphilis originating from other parts of the body, with the most common primary foci found in the skin and mucous membranes of the external genitalia (9). In actual clinical practice, it has been observed that certain patients with CSG exhibit an absence of early symptoms associated with syphilis. Among this cohort, only three patients were considered to have had chancroid symptoms or syphilis rash presentations. One potential explanation is that certain latent syphilis infections may be treated with other antibiotics due to coexisting diseases, leading to changes in the clinical features and classic disease course of neurosyphilis, resulting in an increasing number of atypical neurosyphilitic presentations in clinical practice. However, the development of CSG must be correlated with syphilis, and eight patients were positive for serum TPHA and TRUST. Inadequate treatment of early syphilis, poor compliance, or a lack of awareness of sexually transmitted disease prevention may be one of the important reasons for the development of CSG. The clinical characteristics observed in this cohort were as follows: (1) The prevalence of CSG was higher among middle-aged and elderly males, with patients exhibiting pronounced clinical imaging changes but relatively mild neurological symptoms. (2) Patients initially presented with non-specific headaches, and upon admission, they did not exhibit symptoms of nervous system infection such as fever or meningeal irritation. The absence of characteristic syphilitic skin lesions such as rashes or papules, could lead clinicians to rely solely on routine diagnosis and overlook the necessity of performing a lumbar puncture and laboratory examination of CSF for syphilis in the laboratory. (3) The majority of male patients in this cohort denied a history of promiscuity upon admission, citing concerns over personal privacy. Consequently, obtaining reliable and accurate medical histories proved challenging. (4) There was no significant correlation observed between the serum TRUST titer and the severity of clinical symptoms or the presence/absence of CSG in patients diagnosed with syphilis. This is because we found that the serum TRUST titers of patients with clinically detected CSG did not show high values. (5) The size of CSG lesions did not exhibit a significant correlation with the age of the patients, and there is insufficient evidence to support a correlation with the timing of detection. (6) The majority of cases in this cohort were misdiagnosed as tumor lesions and underwent surgical resection. The imaging findings pose challenges in distinguishing them from brain tumors, indicating a certain level of difficulty in the differential diagnosis based on imaging studies. We hypothesize that the reasons for this result were not only the difference in diagnostic experience but also the diversity of imaging findings in GSC, especially the latter reason.

The utilization of head imaging examination is advantageous in facilitating the diagnosis of CSG. MRI images showed a mixture of low signal or iso-signals on T1-weighted imaging, and high signal, iso-signal, or low signal on T2-weighted imaging (2, 10–12). In the presence of cystic change or necrosis, the signal exhibits a mixed and heterogeneous pattern. On contrast-enhanced MRI, the lesions often showed irregular thick-walled rings or nodular enhancement, and the enhancement pattern may be related to the immaturity of the blood-brain barrier of neovascularization in the inflammatory granulation tissue around the lesion (9–12). In diffusion-weighted imaging (DWI), the non-caseating portion of the lesion presented a mildly elevated signal (13). Literature reports indicated that both perfusion-weighted imaging (PWI) and magnetic resonance spectroscopy (MRS) played a distinctive role in the differential diagnosis of CSG, providing valuable insights into brain metabolism and biochemistry, thereby aiding in distinguishing tumors from inflammatory diseases (14, 15). However, this study is retrospective and none of the patients underwent PWI and MRS examinations. In previous literature reports, CSG was almost always misdiagnosed. Since lesions can manifest various signals on conventional cranial MRI imaging and almost always show significant enhancement, differentiating CSG from other intracranial lesions using MRI imaging techniques alone remains a challenge. Currently, there are also no reports of new imaging techniques being applied to the diagnosis of CSG. In the context of MRI, solitary lesions of CSG need to be differentiated from gliomas, meningiomas, brain abscesses, and tuberculoid granulomas, while multifocal lesions require differentiation from parasitic occupations, intracranial metastases, lymphomas, and other conditions. Due to the presence of significantly enhanced nodules, central necrosis, and extensive surrounding edema in lesions such as glioblastoma, malignant meningioma, inflammatory granuloma, and brain metastases, it is challenging to differentiate CSG from tumors and inflammatory granulomas based solely on imaging findings. A comprehensive assessment that includes cerebrospinal fluid analysis is necessary for accurate diagnosis. However, since CSG typically develops from the meninges, the surrounding meninges will exhibit enhancement, thickening, and the presence of the “meningeal tail sign.” Notably, when the margins of the lesion intersect with the surrounding meninges at obtuse angles, this can provide valuable diagnostic clues.

The preoperative diagnosis of CSG mainly relies on the results of relevant tests for syphilis in CSF. CSF is an important pathological specimen that can be obtained through non-surgical means. The CSF of patients with CSG commonly exhibited an elevated lymphocyte-predominant cell count, increased protein concentration, and normal or slightly decreased glucose and chloride levels (2, 8). According to the literature, routine CSF examination revealed abnormalities in only 65% of confirmed neurosyphilis cases, while serological syphilis testing yields positive results in 62% of case (15, 16). In our study, the CSF samples from all eight patients yielded positive results for both TPHA and TRUST. Therefore, whether CSG cases are necessarily positive for both TPHA and TRUST in the CSF remains to be analyzed in a later stage of a larger statistical summary of data. All patients diagnosed with CSG exhibited positive serum TPHA tests, but the serum TRUST titers were not significantly elevated. Such occurrences were not frequently observed in clinical practice, including several cases we have encountered. This observation suggested that the *Treponema pallidum* was not active in the patient's peripheral blood and did not confirm whether it had breached the blood-brain barrier to cause neurosyphilis. Therefore, in order to mitigate the risk of late-stage neurosyphilis, it is imperative to initiate long-term follow-up and active treponemal treatment for patients with positive serum TPHA and low serum TRUST titers who have not previously undergone treponemal therapy. The feasibility of performing CSF examination for diagnostic purposes, as documented in the literature, diminished in cases where patients exhibit a significant intracranial occupying effect, primarily due to two main reasons. On one hand, the feasibility was reduced due to the presence of symptoms of intracranial hypertension in the majority of patients upon admission, coupled with imaging findings that indicate intracranial occupying effects. In such conditions, lumbar puncture carries certain risks obtaining consent from the patients' families may not always be feasible. On the other hand, the infrequent occurrence of CSG, combined with doctors' limited familiarity with the disease, often results in the oversight of its differential diagnosis. Therefore, lumbar puncture was frequently omitted as a means of CSF examination. Currently, serological syphilis antibody tests have been incorporated into the routine admission screenings for the majority of patients in Chinese hospitals, thereby enhancing diagnostic vigilance among clinicians and radiologists toward CSG to a certain extent. The challenge in clinical diagnosis for patients with a history of *Treponema pallidum* infection lies in establishing the correlation between intracranial occupying lesions and syphilis. In the diagnostic process, it is imperative to consider differentiating CSG when dealing with patients presenting intracranial occupying lesions and positive serum syphilis antibody tests.

Histopathological examination is helpful in the diagnosis of CSG. The gross pathology of CSG presented with a gray-yellow mass, which had a fibrotic capsule, and was tough in texture and elastic. The formation of CSG was a chronic inflammatory process, which was pathologically characterized by a large amount of diffuse inflammatory fibrous tissue proliferation, centered on coagulated caseous necrotic tissue, and surrounded by a larger infiltration of plasma cells and lymphocytes (16). It is speculated that the formation mechanism may involve a delayed hypersensitive reaction of the host toward *Treponema pallidum*, which activates phagocytes to engulf and destroy the syphilis bacteria, leading to inflammatory reactions and granulomatous lesions (17). Furthermore, as a consequence of the diffuse infiltration of inflammatory cells and inflammation of small blood vessels, local tissue may undergo occlusion in small arteries, resulting in ischemia and hypoxia, thereby potentially leading to tissue necrosis (16, 17). The CSG serves as a typical representation of brain granulomatous diseases and especially needs to be differentiated from cerebral tubercular granulomas. CSG exhibits a more severe inflammatory response, therefore, there is a significant increase in fibroblastic hyperplasia, with a prominent infiltration of plasma cells. Additionally, the presence of closed intimal inflammation and perivascular inflammation can be observed. The later stages are characterized by the formation of extensive scarring, with calcification being a rare occurrence. In contrast, the central caseous necrosis in cerebral tubercular granulomas is more complete, and calcification can occur as the disease progresses into a chronic state. Detection of *Treponema pallidum* in pathological tissue poses challenges, whereas mycobacterium tuberculosis is frequently encountered in such specimens (18). Our pathologists did not find the presence of *Treponema pallidum* in the pathological tissues.

Due to the low incidence, non-specific clinical symptoms, diversity of intracranial granulomatous diseases, and clinicians' limited familiarity with the disease, misdiagnosis, and missed diagnosis remain prevalent in clinical settings despite comprehensive imaging evaluation and serological antibody tests. Reaching an accurate diagnosis for CSG, a rare lesion, continues to pose significant challenges. The previous literature indicated that most preoperative diagnoses were misdiagnosed as tumor lesions. The literature indicated that the definitive diagnosis of this disease, as well as the majority of cases in this series, relies on postoperative histopathological examination of the tissue and findings of syphilis in the CSF. Some studies suggest that stereotactic pathological biopsy can confirm CSG as soon as possible (3). However, the number of patients with intracranial lesions combined with syphilis is much higher than the number of CSG patients. In the absence of consideration for CSG as a diagnostic factor, stereotactic pathological biopsy does not constitute a routine approach to establishing a diagnosis. In clinical practice, the diagnosis of this disease requires a comprehensive analysis of clinical manifestations, imaging findings, laboratory tests, and histopathological examination. Furthermore, a comprehensive investigation into the patient's medical history and an intricate physical examination are imperative to enhance the precision of the diagnosis.

The majority of CSG patients can achieve a good clinical prognosis after standard anti-syphilis treatment. Literature review showed that most cases had no recurrence, only a few patients mentioned recurrence, and non-standard detoxification treatment was an important reason for easy recurrence. The treatment recommended by the US Centers for Disease Control and Prevention was used: benzylpenicillin 18–24 million units IV daily, as 3–4 million units every 4 h for 10–14 days (19, 20). After discharge, the relevant serum and cerebrospinal fluid indicators were regularly monitored, and clinical cure was confirmed upon multiple instances of normal indicator levels. Surgical resection of the lesion is indicated only for a select group of CSG patients who exhibit resistance to penicillin or present with a high intracranial pressure crisis (8, 11). Despite undergoing surgery due to preoperative misdiagnosis, all seven patients we treated exhibited a favorable prognosis following standardized postoperative anti-syphilis treatment. Our treatment experience is as follows: (1) Strictly grasping the surgical indications and carrying out anti-syphilis treatment if the condition allows are the basic principles to reduce the unnecessary surgical risks for patients. (2) By utilizing auxiliary positioning systems, such as intraoperative ultrasound and navigation techniques, surgeons can effectively minimize potential damage to brain tissue during surgical procedures. (3) The capsule of the lesion was intact and thick, with a rare occurrence of liquid necrotic pus within the inside. Generally, there is no contamination of the surrounding brain tissue. However, it is necessary to protect the surrounding brain tissue and ensure thorough cleaning of the wound cavity with saline, and usually, there is no need for local antibiotic irrigation. Postoperative standardized anti-syphilis treatment should be initiated as early as possible. (4) In cases presenting with suspicious preoperative diagnoses, whether they are tumor lesions or other diseases, it is imperative to conduct intraoperative rapid frozen pathological examination for definitive diagnosis confirmation, thereby facilitating the selection of an appropriate surgical approach. (5) In cases where obtaining a pathological tissue specimen is not feasible but there exists a strong clinical suspicion of CSG, diagnostic anti-syphilis treatment may be considered if conditions such as malignant intracranial pressure increases, rapid progression of neurological dysfunction, or brain herniation have been ruled out. (6) Several studies evaluated the efficacy of enhanced treatment regimens for syphilis based on the fact that complications may arise despite appropriate penicillin therapy for syphilis (21), We strongly agree with this viewpoint.

## Data Availability

The original contributions presented in the study are included in the article/supplementary material, further inquiries can be directed to the corresponding authors.
